# Identification of a New Conserved Antigenic Epitope by Specific Monoclonal Antibodies Targeting the African Swine Fever Virus Capsid Protein p17

**DOI:** 10.3390/vetsci11120650

**Published:** 2024-12-13

**Authors:** Nengwen Xia, Qi Cao, Anjing Liu, Jiajia Zhang, Hongjian Han, Jun Jiao, Ping He, Ziyan Sun, Zijian Xu, Wanglong Zheng, Sen Jiang, Nanhua Chen, Jianfa Bai, Jianzhong Zhu

**Affiliations:** 1 College Veterinary Medicine, Yangzhou University, Yangzhou 225009, China; 2 Joint International Research Laboratory of Agriculture and Agri-Product Safety, Yangzhou University, Yangzhou 225009, China; 3 Comparative Medicine Research Institute, Yangzhou University, Yangzhou 225009, China; 4 Jiangsu Co-innovation Center for Prevention and Control of Important Animal Infectious Diseases and Zoonoses, Yangzhou University, Yangzhou 225009, China; 5 Kansas State Veterinary Diagnostic Laboratory, Kansas State University, Manhattan, KS 66506, USA

**Keywords:** African swine fever virus, p17, monoclonal antibody, epitope, diagnosis

## Abstract

African swine fever (ASF) has widely spread around the world in the last 100 years since its discovery. The African swine fever virus (ASFV) particles are made of more than 150 proteins, with the p17 protein encoded by the D117L gene serving as one of the major capsid proteins and playing a crucial role in the virus’s morphogenesis and immune evasion. Thus, monoclonal antibody (mAb) targeting p17 is important for the research and detection of ASFV infection. Here, we produced two specific mAbs against p17, designated as 1G2 and 6G3, respectively, and both have been successfully used in enzyme-linked immunosorbent assay (ELISA), Western blotting, and immunofluorescence assay. Moreover, we found that both 1G2 and 6G3 mAbs recognize a novel epitope of 72–78 amino acids of p17 protein, highly conserved across all genotype I and II strains. Based on this epitope, an indirect ELISA has been established to effectively detect antibodies during ASFV infection, and it exhibits high consistency with commercial ASFV ELISA kits. In summary, the production of the specific p17 mAbs and the identification of the recognized epitope will significantly promote the serological diagnosis of ASFV.

## Simple Summary

African swine fever has widely spread in China in the last couple of years and posed a significant impact on pig industry. Currently, there is no safe and effective commercial vaccine available and the prevention and control of the disease mainly rely on the early detection and stamping out strategy. Therefore, it is important to develop a specific and easy for use method for diagnosis of this disease. In this study, we generated two specific monoclonal antibodies against the capsid protein p17 of African swine fever virus particles. Both monoclonal antibodies have been successfully applied in different immune assays including enzyme-linked immunosorbent assay, Western blotting assay and immunofluorescence assay. Moreover, both p17 monoclonal antibodies were found to recognize a new antigenic epitope of 72–78 amino acids of p17 protein, which it is identical across all genotype I and II strains of African swine fever virus. Based on this epitope peptide, an indirect ELISA has been established to effectively detect antibodies during virus infection, and it will significantly promote the serological diagnosis and contribute to the disease prevention and control.

## 1. Introduction

African swine fever (ASF) is a highly contagious and fatal hemorrhagic disease in pigs caused by the African swine fever virus (ASFV). Since its first report in China in 2018, ASF has spread quickly and widely and posed a significant impact on the pig industry in the following years [1,2]. Despite the domestic and international efforts to develop a vaccine, there is currently no effective and safe commercial vaccine available [3,4,5,6]. Therefore, ASF prevention and control mainly rely on rapid detection and early diagnosis.

ASFV is a large double-stranded DNA virus, with a genome of about 170–190 kb, producing over 150 viral proteins [7]. The structure of ASFV is complex and can be divided from the outside towards the inside into the outer membrane, capsid, inner membrane, core-shell, and nucleoid [8]. The p17 protein encoded by ORF D117L is an important component of the ASFV capsid [9]. ASFV p17 plays a critical role in viral morphogenesis, the absence of p17 hinders the proteolysis of the viral polyproteins pp220 and pp62, thereby affecting the assembly of the viral particles [10].

ASFV p17 not only affects the morphogenesis and assembly of viral particles but also participates in the regulation of host cell function and immune evasion. In eukaryotic cells, p17 is located on the endoplasmic reticulum (ER) membrane and its expression can cause ER stress, leading to the release of reactive oxygen species, which inhibits cell proliferation by arresting the cell cycle [11]. The interaction of p17 with TOMM70 promotes the binding of the autophagy receptor SQSTM1 with TOMM70, resulting in mitophagy and decreased expression of MAVS, thereby suppressing the innate immune response [12]. The p17 is involved in the regulation of innate immunity mediated by the cGAS-STING signaling cascade. On the one hand, p17 binds to STING, inhibiting the interactions between STING and TBK1/IKKε, and blocking the induction of type I IFN [13]. On the other hand, p17 downregulates the TBK1 and IRF3 phosphorylation by recruiting the host scaffold protein PR65A and triggering STING partial degradation via apoptosis [14].

As one of the main capsid proteins, p17 has a high abundance in ASFV particles and possesses a good immunogenicity [7], making it a potential target for detecting ASFV infection. There has been a report of p17 expression in CHO cells and the establishment of a specific indirect ELISA based on the purified p17 from CHO cells [15]. The p17 monoclonal antibodies (mAbs) were generated from eukaryotic p17 protein-immunized mice and the mAb recognized linear epitopes are located at the N-terminus of p17 [15,16]. In this research, we expressed and purified the bacterial p17 to produce p17 mAbs from immunized mice. Two p17 mAbs generated could recognize a conserved linear epitope at 72–78 amino acids in the middle of p17. Additionally, the epitope indirect ELISA was established, and it was capable of specifically detecting ASFV antibodies in the serum samples of pigs with ASF. Our results provide useful tools for ASFV scientific research but also establish a new assay for detection and prevention of ASF.

## 2. Materials and Methods

### 2.1. Mice, Cells, Viruses, Sera and Reagents

The 6–8 weeks old BALB/c mice were from the Laboratory Animal Center of Yangzhou University. The animal operation was strictly in accordance with the Guidance for the Care and Use of Laboratory Animals of Yangzhou University (SYXK(JS)-2021-0026). HEK-293T cells (ATCC Cat # CRL-3216), Marc-145 cells (Cellosaurus Cat # CVCL_4540), Vero cells (ATCC Cat # CCL-81), PK-15 cells (ATCC Cat # CCL-33), MDCK cells (ATCC Cat # CCL-34), and myeloma cell line SP2/0 (ATCC Cat # CRL-1581) were cultured in Dulbecco modified Eagle medium (DMEM, Hyclone Laboratories Inc, Omaha, USA) containing 10% fetal bovine serum (FBS) and 100 IU/mL of penicillin plus 100 μg/mL streptomycin. Primary porcine alveolar macrophages (PAMs) and 3D4/21 cells (ATCC cat# CRL-2843) were both cultured in RPMI 1640 medium (Hyclone Laboratories) containing 100 IU/mL of penicillin plus 100 μg/mL streptomycin and 10% FBS. All cells were grown at 37 °C in a 5% CO_2_ humidified incubator. The ASFV used (genotype II, GenBank accession ON456300) was stored in the animal biosafety level 3 (ABSL-3) of Yangzhou University approved by the Ministry of Agriculture and Rural Affairs (07140020201109-1), whereas the porcine reproductive and respiratory syndrome virus (PRRSV), recombinant PRRSV expressing p17 (PRRSV-p17), porcine epidemic diarrhea virus (PEDV), porcine delta coronavirus (PDCoV), swine influenza virus (SIV), and porcine serum samples were kept in our BSL2 lab. The African swine fever virus ELISA antibody detection kit (Cat# 163698918) was bought from Putai Biology Technology Co., Ltd.(Luoyang, China) The RFP mAb (ab185921) was bought from Abcam (Cambridge, UK). The anti-β-actin mAb (5057) was purchased from Cell Signaling Technology (Boston, MA, USA). The anti-PRRSV N mAb, anti-PEDV N mAb, and anti-SIV NP mAb were produced and stored in our lab. The anti-PDCoV NS7 mAb was a gift from Prof. Zhenhai Chen of Yangzhou University.

### 2.2. Expression and Purification of p17 Protein

The ASFV p17 C-terminal intramembrane region (61–171 aa) gene coding sequence D117L was amplified by PCR from pCAGGS-p17-HA stored in our laboratory and cloned into *Xho* I and *EcoR* I sites of pCold-TF vector or *Sal* I and *Xba* I sites of pCold-MBP vector by Seemless/In-Fusion Cloning (Abclonal, Wuhan, China). In the vectors, both tags TF (trigger factor) and MBP (maltose binding protein) are two molecular chaperone proteins that promote the soluble expression of the target protein. The plasmids were transformed into BL21/DE3 *E. coli* competent cells and treated with 1 mM isopropyl-β-d-1-thiogalactoside (IPTG) at 16 °C or 37 °C for 24 h to induce p17 expressions. The expressed p17 fusion proteins were purified by the gel recovery method. Specifically, the bacteria pellet was collected after centrifugation, resuspended in PBS, and sonicated on ice. Following centrifugation, the supernatant was retained and run in SDS-PAGE as much as possible. After staining with Coomassie brilliant blue and subsequent complete de-staining, the target gel piece was cut out and the ground gel was placed in a dialysis bag for electrophoresis at 120 V for 2 h. Finally, the liquid in the dialysis bag was transferred and dialyzed in PBS overnight at 4 °C, and concentrated with PEG 20000.

### 2.3. Production of Anti-p17 Monoclonal Antibodies (mAbs)

Firstly, BALB/c mice were subcutaneously immunized at multiple points on the back with 100 μg purified TF-p17 protein mixed with Montanide gel (SEPPIC SA Cedex, la Garenne Colombes, France) at a volume ratio of 10:1. The second and third immunizations were performed at 14 d and 21 d, respectively, after the first immunization, with each 50 μg protein mixed with same adjuvant. Five days later, tail vein blood was drawn and serum p17 antibody was measured by p17 protein indirect ELISA. Mic exhibiting the highest antibody level was selected for the last boost (50 μg p17 protein injection intraperitoneally without adjuvant). Subsequently, the mice were sacrificed three days later, and the spleen cells were collected and fused with SP2/0 cells at a ratio of 5–10:1 using PEG1500 following the standard procedure. The fused cells in 96-well cell culture plates containing peritoneal feeder cells were cultured and selected in the HAT medium in a 37 °C, 5% CO_2_ incubator. The growth of the hybridoma cells was observed, and the hybridomas secreting p17-specific antibodies were screened out by MBP-p17-based indirect ELISA, followed by Western blotting confirmation. Positive hybridomas were subjected to limited dilution subcloning three times and tested further for antibody secretion.

### 2.4. Mapping of the Precise Linear B Cell Epitope of p17 Protein

Strategically, the C-terminal intramembrane region of p17 (aa 61–117) was first separated into three fragments which were tested for reactivity with p17 mAbs. Next, based on information on the reacted fragment, progressive truncations at both C terminal ends were performed on full-length p17. The reactivity of different truncated fragments with p17 mAbs was tested, the critical amino acids of both ends for reactivity with p17 mAbs were determined, and the minimal epitope was thus deduced. The template pCAGGS-p17-HA and pDsRed-C1-p17 plasmids were previously constructed and used in this study [11,13]. In total, 14 p17 fragments (P1-P14) were designed, with all cloning PCR primers listed in Table S1. All p17 fragments were amplified by PCR using the template pCAGGS-p17-HA and then cloned into the *Bgl* II and *EcoR* I sites of pDsRed-C1-express vector by Seemless/In-Fusion Cloning. All the constructed plasmids were transfected into 293T cells, and the reactivity of different truncated p17 proteins with p17 mAbs was tested using Western blotting.

### 2.5. Western Blotting

The reactivity of anti-p17 mAbs with p17 expressed in transfected 293T cells, PRRSV-p17 infected Marc-145 cells, and ASFV infected PAMs, as well as the p17 fragments in transfected 293T cells, were evaluated using Western blotting. The cell total proteins were run and separated by 6–10% SDS-PAGE, and next transferred to PVDF membranes. The membranes were then blocked with 5% non-fat dry milk TBS solution with 0.1% Tween-20 (TBST) for 1 h. Next, the PVDF membrane was probed with the primary mAbs (1:1000 anti-RFP and p17 hybridoma ascites) at 4 °C overnight. Next, the membrane was probed with HRP-conjugated Goat Anti-Mouse IgG (1:10,000, BBI, Shanghai, China) for 1 h. Protein signals were detected with the enhanced ECL substrate (Tanon, Shanghai, China), and visualized and captured by a Western blot imaging system (Tanon, China).

### 2.6. Immunofluorescence Assa

The 3D4/21 cells were transfected with pCAGGS-p17-HA or pdsRed-C1-p17/truncated mutants for 24 h, while Marc-145 cells were infected with PRRSV-p17 with a multiplicity of infection (MOI) of 0.1 for 72 h. The cells were then fixed in 4% paraformaldehyde for about 30 min, permeabilized with 0.5% Triton X-100, and blocked in 5% BSA. Treated cells were probed with p17 mAbs (1:200 ascites) overnight, and then Donkey anti-Mouse IgG (H + L) Alexa Fluor 488 (1:500, Invitrogen, Shanghai, China) for 1 h, which was followed by DAPI staining (Beyotime, Shanghai, China). Cells were finally examined and visualized by fluorescence microscopy at the excitation wavelengths 405 nm, 488 nm, and 535 nm, respectively.

### 2.7. Enzyme-Linked Immunosorbent Assay

For p17 protein indirect ELISA, the ELISA plate wells were coated with p17 protein (MBP-p17) in PBS with the concentration of 0.3125 μg/mL at 4 °C overnight, followed by washing and blocking of 5% skim milk TBST at 37 °C for 2 h. Hybridoma supernatant (100 μL/well) was dispensed to each well and reacted for 2 h at 37 °C. Following washing with PBST, Goat anti-Mouse IgG-HRP (1:10,000 dilution, 100 μL/well, TransGen Biotech, Beijing, China) was incubated at 37 °C for 1 h. Next, TMB (50 μL/well) was added and reacted for 15 min at 37 °C in the dark. The reaction was terminated with 0.5 M H_2_SO_4_ and the optical density at 450 nm (OD_450_) was detected. The ratios of hybridoma cell supernatants to SP2/0 supernatant (P/N, ratio of positive to negative) were calculated, with P/N ≥ 2.0 considered as positive.

For p17 epitope indirect ELISA, ELISA plate wells were coated with antigenic epitope peptide in PBS at the concentration of 0.3125–10 μg/mL at 4 °C overnight, followed by washing and blocking of 5% BSA at 37 °C for 2 h. Diluted porcine serum (1:5–1:800, 100 μL/well) was incubated at 37 °C for 2 h. After washing with PBST, secondary antibody Goat anti-Swine IgG-HRP (1:10,000, 100 μL/well, Proteintech, Wuhan, China) was incubated at 37 °C for 1 h, followed by substrate TMB incubation, stopping of 0.5 M H_2_SO_4,_ and measurement of OD_450_. The ratios of positive sera to normal serum (P/N) were examined

### 2.8. Bioinformatics Analysis

The ASFV p17 protein transmembrane (TM) was analyzed and predicted by online software TMHMM-2.0 (https://services.healthtech.dtu.dk/services/TMHMM-2.0/ (accessed on 9 December 2024)). The analysis of hydrophobicity or hydrophilicity scales of p17 was performed by the online software ProtScale (https://web.expasy.org/protscale/ (accessed on 9 December 2024)). The B cell epitopes in the p17 protein were predicted via the online tool (http://tools.iedb.org/main/bcell/ (accessed on 9 December 2024)). To verify the consensus of the identified p17 epitope, the p17 amino acid sequences of 207 ASFV strains from GenBank were downloaded. The alignment of amino acid sequences and conservation analysis was performed by DNAMAN software, version 9.0 (San Ramon, CA, USA). The spatial distribution and structure of epitope in p17 protein was predicted by Alphafold2 (https://colab.research.google.com/github/sokrypton/colabFold/blob/main/Alphafold2.ipynb (accessed on 9 December 2024)) and visualized by the PyMOL Molecular Graphics System (Version 2.4.0, Schrödinger, LLC, New York, USA).

### 2.9. Quantification and Statistical Analysis

Statistical analyses were carried on by GraphPad Prism 8 software (GraphPad Software, Inc., San Diego, CA, USA) with the data presented as means ± SEM (*n* = 3). The *p* values were calculated by using an unpaired *t* test with normality determined by Shapiro–Wilk. Statistical significance was represented as not significant (NS): *p* > 0.05, *: *p* ≤ 0.05, and **: *p* ≤ 0.01. The *p* ≤ 0.05 was considered as statistically significant.

## 3. Results

### 3.1. Characterization and Production of Recombinant p17 Protein

**Figure 1:**
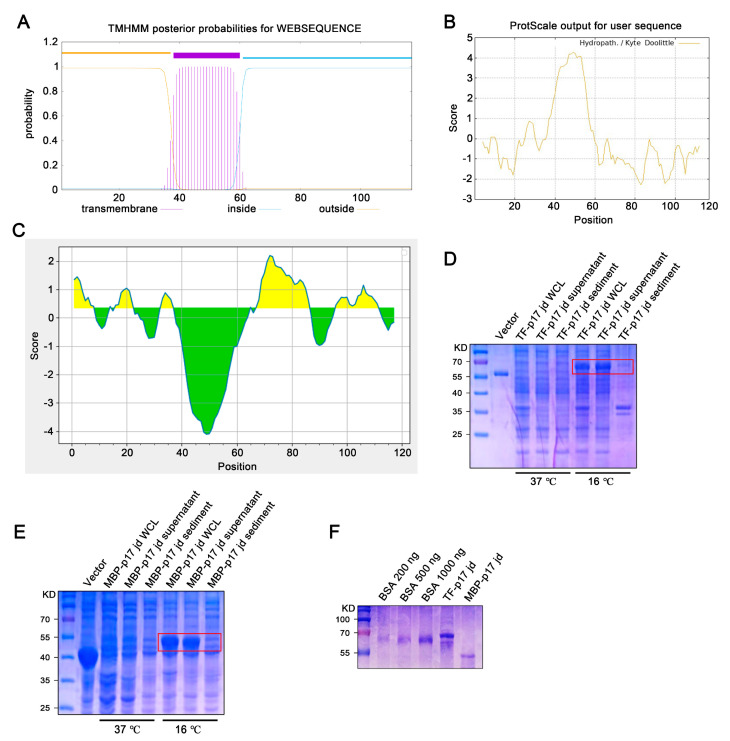
Production and identification of the p17 truncated fusion proteins. (**A**–**C**) Analysis of the transmembrane region (**A**), hydrophilicity (**B**), and B cell epitopes colored in yellow (**C**) of p17 protein using online tools, as described in Methods. In panel C, the yellow and green colors represent plus and minus scores, respectively, whereas the higher the score, the higher probability of being part of epitope. (**D**,**E**) The TF-p17 jd (**D**) and MBP-p17 jd (**E**) were induced by IPTG at a final concentration of 1 mM at 37 °C and 16 °C. Whole-cell lysate, supernatant, and precipitate were examined for p17 protein expressions by SDS-PAGE plus Coomassie blue staining, with the major band about 68 kD (**D**) and 53 kD (**E**) which are red-boxed. (**F**) The purified p17 fusion proteins were verified by SDS-PAGE plus Coomassie blue staining against standard bovine serum albumin (BSA). Based on the band density relative to BSA bands, the concentration of TF-p17 jd protein is estimated greater than 1 μg/μL, while the concentration of MBP-p17 jd protein is approximately 500 ng/μL.

Bioinformatic analysis showed that the ASFV p17 protein is a transmembrane protein, with the transmembrane (TM) region located at 38–60 amino acids (aa) (Figure 1A), which corresponds to the highly hydrophobic region (Figure 1B). Both the extramembrane region (1–37 aa) and intramembrane region (61–117 aa) contain the B cells antigenic epitopes highlighted in yellow color (Figure 1C). Given that the C terminal intramembrane region contains more antigenicity (Figure 1C), this hydrophilic region (61–117 aa) was selected for cloning and fusion expression with soluble tags, *E. coli* chaperone proteins trigger factor (TF), and maltose binding protein (MBP), respectively. The constructed recombinant prokaryotic expression plasmids, pCold-TF-p17 jd and pCold-MBP-p17 jd, were transformed into DE3/BL21 competent *E. coli* and cultured at 37 °C or 16 °C with the induction of 1 mM IPTG for 24 h. The soluble expressions of both TF-p17 jd (Figure 1D) and MBP-p17 jd (Figure 1E) were significantly induced by IPTG at 16 °C rather than 37 °C. SDS-PAGE analysis showed that the purified TF-p17 jd and MBP-p17 jd proteins had high purity, and exhibited the molecular weights of approximately 68 kD and 53 kD, respectively, as expected (Figure 1F).

### 3.2. Production of Specific Monoclonal Antibodies (mAbs) of p17 Protein

**Figure 2:**
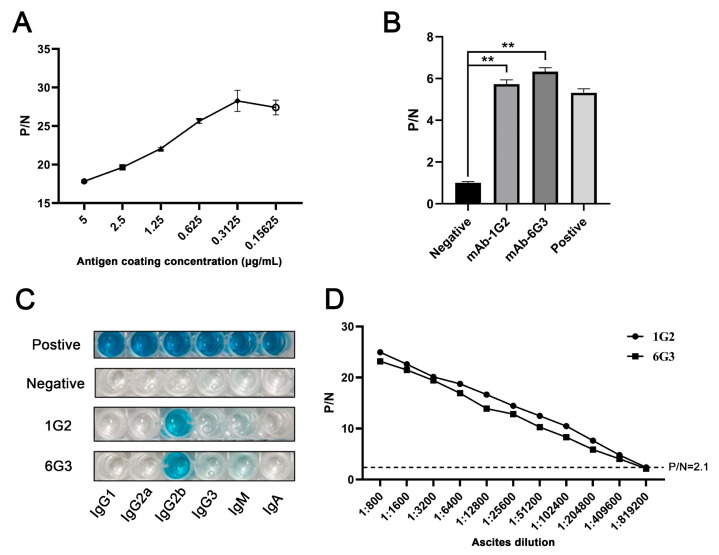
Characterization of anti-p17 mAbs. (**A**) Optimization of the amount of coating MBP-p17 jd protein in indirect ELISA detecting ASFV positive serum. (**B**) The mAbs were tested in MBP-p17 indirect ELISA. The supernatants of hybridoma 1G2 and 6G3 were used as the primary antibodies, the SP2/0 supernatant was used as the negative control, and the serum from immunized mice was used as positive control. ** *p* < 0.01. (**C**) Subclass of p17 mAbs 1G2 and 6G3 was determined by the monoclonal antibody subclass identification kit (C060101) from CELLWAY-LAB (Luoyang, China). (**D**) Titer measurement of the ascite mAbs 1G2 and 6G3 using MBP-p17 protein indirect ELISA. The dotted line represents the P/N value of 2.1.

**Figure 3:**
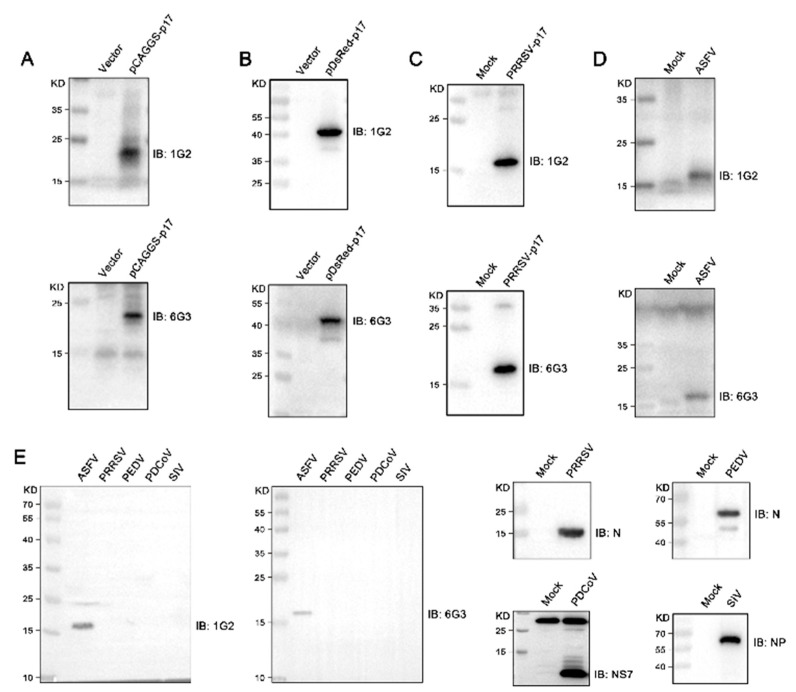
Analysis of the reactivity of p17 mAbs using Western blotting. (**A**–**C**) 293T cells were transfected with pCAGGS-p17-HA (1 μg/mL) (**A**) and pDsRed-p17 (1 μg/mL) (**B**) with pCAGGS vector and pDsRed vector as the controls. Marc-145 cells were infected with PRRSV-p17 with a multiplicity of infection (MOI) of 0.1 for 72 h (**C**). Cells were harvested and cell lysates were detected for exogenous p17 using Western blotting with 1G2 and 6G3 mAbs as primary antibodies. (**D**) Primary PAMs were infected with ASFV (0.1 MOI) or mock-infected for 96 h, and cell lysates were examined for endogenous p17 using Western blotting with the mAbs 1G2 and 6G3 as primary antibodies. (**E**) Primary PAMs, Marc-145 cells, Vero cells, PK15 cells, and MDCK cells were infected with ASFV (MOI 0.1), PRRSV (MOI 0.1), PEDV (MOI 0.1), PDCoV (MOI 0.1) and SIV (MOI 0.1), respectively. Cells were harvested and cell lysates were detected the specificity of p17 mAbs by Western blotting with 1G2 and 6G3 mAbs as primary antibodies. The replications of PRRSV, PEDV, PDCoV, and SIV were detected using Western blotting with the antibodies indicated. The original images of the Western blot are published as Supplementary Materials.

The hybridomas were acquired by standard method for cell fusion between spleen cells of mice immunized with TF-p17 jd and myeloma SP2/0 cells, and positive hybridoma clones were screened out by MBP-p17 protein indirect ELISA as stated in Section 2.7. We determined the optimal coating concentration of p17 protein using ASFV-positive pig serum as 0.3125 μg/mL (Figure 2A). After screening by indirect ELISA and three rounds of subcloning using a limited dilution method, two pure hybridoma cell clones named 1G2 and 6G3 were generated (Figure 2B). The monoclonal antibodies (mAbs) produced by both 1G2 and 6G3 clones are both the IgG2b subclass (Figure 2C). The titers of ascites 1G2 and 6G3 mAbs were measured by ELISA to be 400,000–800,000 (Figure 2D). The specific reactivity of the two mAbs with the eukaryotic expressed p17 proteins from 293T cells transfected with pCAGGS-p17-HA (Figure 3A) and pDsRed-p17 (Figure 3B), from Marc-145 cells infected with PRRSV-p17 (Figure 3C) and from primary PAMs infected with ASFV (Figure 3D) was determined using Western blotting as stated in Section 2.5. These results illustrated that both 1G2 and 6G3 specifically react with not only exogenous p17 (Figure 3A–C) but also endogenous p17 (Figure 3D).

**Figure 4:**
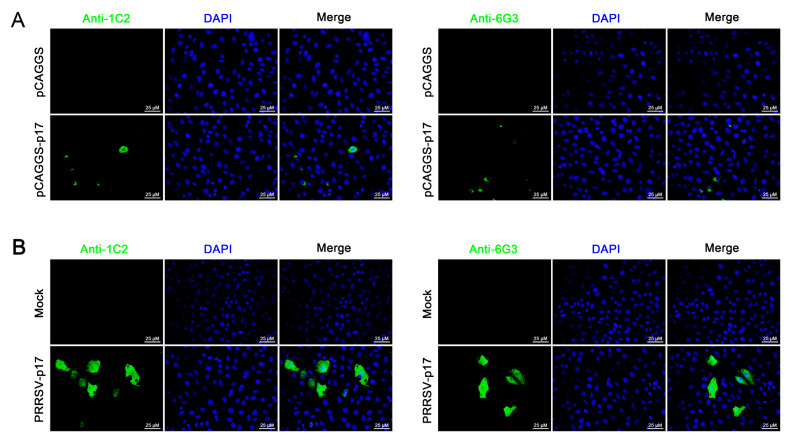
Analysis of specific reactivity of p17 mAbs by Immunofluorescence. (**A**) 3D4/21 cells were transfected with pCAGGS-p17-HA and pCAGGS control vectors, respectively. Cells were fixed at 24 h post-transfection and stained with 1G2 or 6G3 mAbs, plus Goat anti-mouse IgG H&L Alexa Fluor 488. Cellular nuclei were counterstained with DAPI. (**B**) Marc-145 cells were infected with PRRSV-p17 (MOI 0.1). Cells were fixed at 72 h post-infection and stained with 1G2 or 6G3, plus secondary antibody and DAPI.

Further tests were conducted to assess the reactivity of the two mAbs with various swine viruses. The results showed that both 1G2 and 6G3 only reacted with AFSV-infected samples, but not other porcine viruses including PRRSV, PEDV, PDCoV, and SIV (Figure 3E). Similarly, immunofluorescence analysis indicated that both mAbs could react specifically with the p17 expressed in transfected 3D4/21 cells (Figure 4A and Figure S1) and in PRRSV-p17-infected Marc-145 cells (Figure 4B), with the detected p17 primarily located in the cytoplasm. These results collectively demonstrated that the produced mAbs are specific to ASFV p17, successfully detecting p17 in various immune assays.

### 3.3. Identification and Bioinformatics Analysis of the Epitope Recognized by Two p17 mAbs

**Figure 5:**
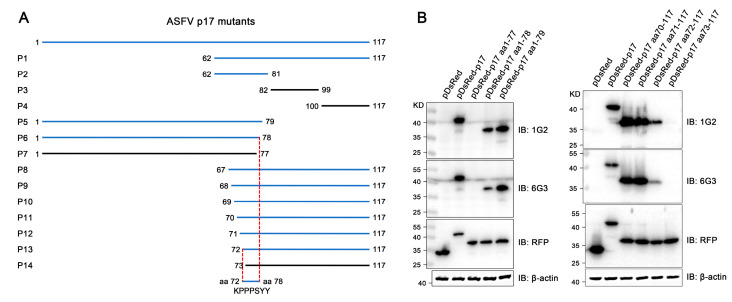
Precise mapping of the antigenic epitopes recognized by p17 mAbs. (**A**) Schematic for precise mapping of the epitope. The fragments with reactivity with p17 mAbs are blue marked, whereas those of no reactivity are black marked. (**B**) Western blotting analyzed the roles of critical C terminal amino acid (**left**) and N terminal amino acid (**right**) in reactivity of p17 proteins with the two mAbs 1G2 and 6G3. The aa denotes the abbreviation for amino acid. The original images of the Western blot are published as a Supplementary Material.

The precise mapping of antigenic epitopes for two mAbs was conducted by segmental and gradient truncation of the p17, each of which was cloned into the pDsRed-C1 expression vector (Figure 5A). Western blotting results showed that the deletion of N-terminal K72 and C-terminal Y78 would disrupt the reactivity of two mAbs with p17 protein, indicating that the epitope recognized by the two p17 mAbs is ^72^KPPPSYY^78^ (Figure 5B and Figure S2). Similarly, in the immunofluorescence assay, the deletion of K72 or Y78 in p17 completely abolished the reactivity with the mAbs (Figure S3). As such, the results clearly demonstrated that both 1G2 and 6G3 mAbs recognize the linear epitope ^72^KPPPSYY^78^.

**Figure 6:**
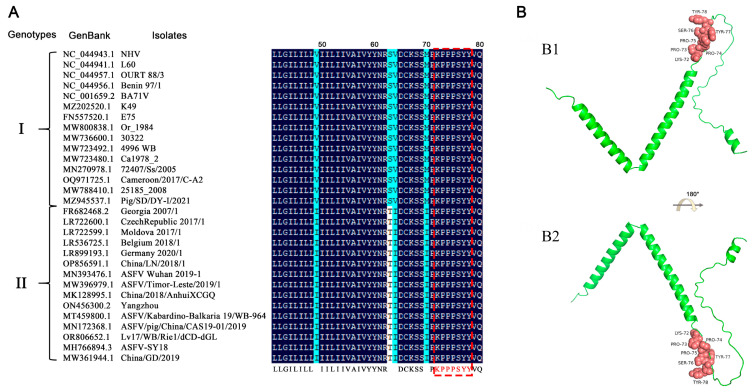
Conservation of the identified novel linear epitope of the p17 protein. (**A**) Alignment of the epitope (^72^KPPPSYY^78^) of 30 representative genotypes I and II ASFV strains. The red box indicated the identified epitope. (**B**) Prediction of the p17 structure using Alphafold2 online web tools and visualization with PyMOL. The epitope of the two mAbs is displayed in pink color and shown in the front view (**B1**) and in flipping 180° along the *x*-axis (**B2**), respectively.

To assess the conservation of the antigenic epitope across different ASFV strains, the p17 protein sequences of 207 ASFV strains were extracted from GenBank and compared with the identified antigenic epitope sequence. The results showed that the amino acids 72–78 of all genotypes I and II ASFV p17 are identical, some of which are presented as representatives (Figure 6A). However, the amino acid sequences in other genotypes of ASFV p17 proteins have differences, hence exhibiting only relative conservation (Table S2). Due to the lack of precise crystal information for ASFV p17 protein in the Protein Data Bank (PDB), we utilized the Alphafold2 web tools to predict the tertiary structure of the p17 protein, followed by visualization with PyMOL. The results indicated that the antigenic epitope of the two mAbs is located at the junction of an alpha-helix and a random coil (Figure 6B).

### 3.4. Establishing the Epitope ELISA for Detection of ASFV Antibodies

**Figure 7:**
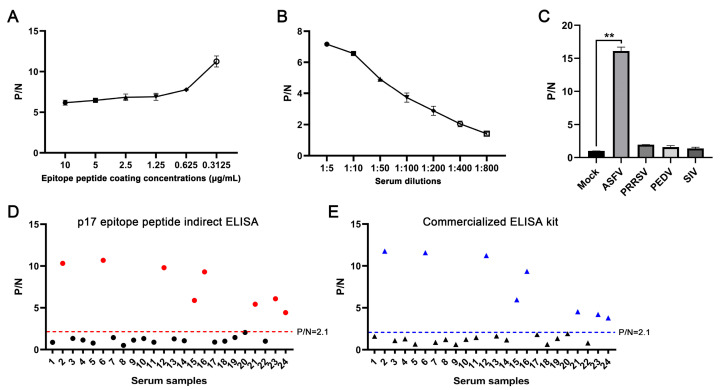
Establishment of epitope-based indirect ELISA to detect ASFV antibodies. (**A**) The p17 epitope peptide (synthesized by GeneCreate Wuhan, China) was used for coating at concentrations from 0.3125–10 μg/mL. The epitope ELISA was used for detection of ASFV-positive serum (1:5 dilution) and negative normal pig serum to determine the optimized concentration of coating epitope peptide. (**B**) Optimization of serum dilution with peptide coating concentration at 0.3125 μg/mL. (**C**) The specificity of the established epitope ELISA. PRRSV, PEDV, SIV, and ASFV positive pig sera and negative pig serum were used. ** *p* < 0.01. (**D**,**E**) 24 clinical pig sera were tested with our established indirect epitope peptide ELISA (**D**) and the commercial ASFV ELISA detection kit (**E**), with pig-negative serum as the control. The samples marked in red (**D**) and blue (**E**) are those with positive detection results.

In order to verify the diagnostic ability of the epitope recognized by the p17 mAbs, we developed an epitope indirect ELISA to detect the ASFV antibodies as stated in Section 2.7. The first optimization of the indirect ELISA showed that the highest positive-to-negative (P/N) ratio was achieved when the p17 epitope peptide was coated at a concentration of 0.3125 μg/mL for the detection of ASFV-positive serum (Figure 7A). Second, the dilution of ASFV-positive serum at 1:5 yielded the highest P/N ratio (Figure 7B). The specificity of the epitope indirect ELISA was tested with different positive serum samples of PRRSV, PEDV, SIV, and ASFV. The ELISA results showed that only ASFV-positive serum, rather than others, give a positive P/N ratio (Figure 7C). Our developed epitope ELISA was applied for the detection of 24 pig clinical serum samples, and 9 sera were detected as positive (Figure 7D). In comparison, a commercial ELISA kit was also used for parallel detection, and both detection methods achieved a 100% consistency of positive sample detection rate (Figure 7D,E). Therefore, our preliminary results suggested that the epitope recognized by the p17 mAbs can be used in indirect ELISA to detect ASFV infection.

## 4. Discussion

African Swine Fever (ASF) has a history of over 100 years since its first report in Kenya in 1921 [1]. Over the following decades, ASF made the leap from Africa to Europe, spreading widely until it entered China in 2018 [2]. It is well known that China is a major country for pig farming and product consumption, with pig farming and inventory accounting for more than half of the global total [17]. The prevalence of ASF in China has caused the mutation and recombination of the ASFV [18,19]. At present, the recombinant ASFV of genotypes I and II have been discovered in China, some of which, although classified as genotype I, have inserted multiple virulence genes of genotype II and exhibit high virulence and transmissibility in pigs [18]. These pose a huge challenge to ASF prevention and control.

The inactivated vaccine for ASF has shown poor protective efficacy [20], and the gene-deleted vaccine has been observed to undergo gene recombination in the field, carrying the risk of virulence reversion [19,21]. For ASF prevention and control, detection has become a crucial procedure [22]. Currently, the detection of ASF primarily focuses on the detection of the viral genome or particles, as well as the antibodies related to ASFV [23]. In subacute and chronic infections, serological detection of ASFV-specific antibodies is a pretty reliable means [23]. The production of mAbs against ASFV structural proteins, the identification of antigenic epitopes, and the subsequent establishment of detection methods mainly concentrated on structural proteins such as p72 [24,25,26,27], p54 [28,29,30,31,32], p30 [33,34,35], and CD2v [36,37,38], with only a few reports on p17. ASFV p17 closely surrounds p72 in a trimeric form and is crucial for the assembly of the ASFV capsid and the formation of the icosahedral morphology [9]. Besides mediating viral morphogenesis and immune evasion, the expression of p17 also affects various host cell events, including host cell endocytosis, ubiquitin-related proteolysis, N-glycan-mediated biosynthesis, and cell apoptosis [39].

Due to the poor prokaryotic expression of full-length p17 (not shown), we discarded the hydrophobic transmembrane region and selected the hydrophilic intramembrane region for fusion with two molecular chaperone proteins, TF and MBP, respectively (Figure 1). By immunizing with TF-p17 protein and screening of the hybridomas by MBP-p17 based indirect ELISA to reduce the non-specificity, we obtained two p17 specific mAbs, which work successfully in multiple immune experiments including ELISA, Western blotting, and Immunofluorescence (Figure 2, Figure 3, Figure 4 and Figure S1), demonstrating the value of broad application. Notably, the mAb-recognized p17 in transfected cells and PRRSV-17 infected cells are about 23 kD, whereas the mAb-recognized p17 in ASFV-infected cells is about 17 kD (Figure 3A). The size difference of p17 in different expression systems indicated the disparity of post-translational modification of the p17 protein under different conditions, which is interesting and deserves further investigation.

We identified the minimal antigenic epitope recognized by these two p17 mAbs that is ^72^KPPPSYY^78^ (Figure 5, Figures S2 and S3). Previous studies identified two antigenic epitopes in p17 that are ^3^TETSPLLSH^11^ and ^8^LLSHNLSTREGIK^20^, by using p17 mAbs [15,16]. Consistent with our study, a recent study predicted and confirmed that ^63^TIDCKSSIPKPPPSYYVQQPEPHH^86^ is one of the most immunogenic and immunoreactive epitopes [40], further confirming the validity of this antigenic epitope. The identification of precise antigenic epitope justified the utilization of the epitope in the serological determination of ASFV infection. From the highly conserved antigenic epitope recognized by p17 mAbs (Figure 6), we established an indirect ELISA to detect the antibodies against ASFV (Figure 7). In the detection of 24 clinical serum samples from pigs, 9 of 24 serum samples were detected as positive (Figure 7D). The results were totally consistent with that of the commercial ELISA antibody detection kit as stated in Materials (Figure 7E), suggesting the effectiveness and validity of our peptide ELISA assay and the potential for practical application in the clinic. Given the purity and consistency of synthesized epitope as the coating antigen, the peptide ELISA may provide certain advantages or at least an alternative to the commercial ELISA kit for the detection of ASFV antibodies. Recently, our team has developed both RPA-LbCas12a and RPA-LwCas13a detection methods on the ASFV capsid structural gene (D117L), reaching a sensitivity as high as two gene copies, showing no cross-reactivity with other common pig viruses [41,42]. Thus, the combined detections of nucleic acid and antibodies based on the ASFV structural D117L gene/p17 protein can greatly improve the detection of ASFV infection, achieving convenience and precision.

Collectively, we generated two specific mAbs of ASFV p17, identified a highly conserved B-cell epitope of p17, and established an epitope-based indirect ELISA detecting ASFV antibodies. These tools not only provide effective means for in-depth research on the structural p17 protein of ASFV but also contribute to ASF prevention and control.

## Data Availability

All data are available in the article.

## Supplementary Materials

The following supporting information can be downloaded at: https://www.mdpi.com/article/10.3390/vetsci11120650/s1, Figure S1: Analysis of specific reactivity of p17 mAbs by Immunofluorescence. 3D4/21 cells were transfected pDsRed-p17 (1 μg/mL) and pDsRed-C1 vector, respectively. Cells were fixed at 24 h post-transfection and stained with 1G2 mAbs (A) and 6G3 (B), together with Goat anti-mouse IgG H&L Alexa Fluor 488. Cellular nuclei were counterstained with DAPI. The reactivity of p17 with mAbs was visualized by fluorescence microscopy; Figure S2: The reactivity of different p17 truncated mutants with p17 monoclonal antibodies. Western blotting analysis of the truncated p17 mutants as indicated in (A,B) with both p17 mAbs 1G2 and 6G3. All mutants were also examined for tag RFP expression using an anti-DsRed antibody. The aa is an abbreviation for amino acid; Figure S3: Immunofluorescence identification of antigenic epitope recognized by p17 mAbs. 3D4/21 cells were transfected pDsRed-p17 (1 μg/mL) and mutants, respectively. Cells were fixed at 24 h post-transfection and stained with mAbs 1G2 (A) or 6G3 (B), together with Goat anti-mouse IgG H&L Alexa Fluor 488. Cellular nuclei were counterstained with DAPI. The reactivity of p17 mutants with mAbs was observed by fluorescence microscopy. The boxed areas of merged images were magnified and placed on the upright or upleft corners. The p17 and p17 1–78 are mainly expressed in the cytoplasm, whereas the p17 72–117 is expressed in whole cells; Table S1: PCR Primers for porcine gene mutation and cloning; Table S2: The identity of p17 epitope across different ASFV stains.
