# Supplementary material for: Identification of a New Conserved Antigenic Epitope by Specific Monoclonal Antibodies Targeting the African Swine Fever Virus Capsid Protein p17
Source: Vet Sci. 2024 Dec 13;11(12):650. doi: 10.3390/vetsci11120650 (PMC11680328; doi:10.3390/vetsci11120650)
Supplement: Supplementary file 1 [file vetsci-11-00650-s001.zip › Supplemental Table S1.pdf]

Supplementary Table S1. PCR Primers for porcine gene mutation and cloning

Primers	Sequence (5'-3')
**pCold-TF-p17-F**	5'-ATGGGTACCCTCGAGCGGACTATTGACTGCAAGTCGA-3'
**pCold-TF-p17-R**	5'-GTCGACAAGCTTGAATTCTTATGAATGCGCAAGTTCAGCT-3'
**pCold-MBP-p17-F**	5'-TCCGAATTCAAGCTTGTCGACCGGACTATTGACTGCAAGTCG-3'
**pCold-MBP-p17-R**	5'-AGCAGAGATTACCTATCTAGATGAATGCGCAAGTTCAGCTAA-3'
**pDsRed-P1/P2-F**	5'-CACCACCTGTTCCTGAGATCTCGGACTATTGACTGCAAGTCG-3'
**pDsRed-P1/P4/P8-P14-R**	5'-GTACCGTCGACTGCAGAATTCTTATGAATGCGCAAGTTCAGC-3'
**pDsRed-P2-R**	5'-GTACCGTCGACTGCAGAATTCTTATTGTTGTACATAGTAGCT-3'
**pDsRed-P3-F**	5'-CACCACCTGTTCCTGAGATCTCCTGAGCCTCACCACCATTTC-3'
**pDsRed-P3-R**	5'-GTACCGTCGACTGCAGAATTCTTAGGTGGAGTTTTTCCTTTT-3'
**pDsRed-P4-F**	5'-CACCACCTGTTCCTGAGATCTTCCCTGCAGTCCCACATTCCA-3'
**pDsRed-P5/P6/P7-F**	5'-CACCACCTGTTCCTGAGATCTATGGACACTGAAACGTCTCCA-3'
**pDsRed-P5-R**	5'-GTACCGTCGACTGCAGAATTCTTATACATAGTAGCTAGGAGG-3'
**pDsRed-P6-R**	5'-GTACCGTCGACTGCAGAATTCTTAATAGTAGCTAGGAGGAGG-3'
**pDsRed-P7-R**	5'-GTACCGTCGACTGCAGAATTCTTAGTAGCTAGGAGGAGGTTT-3'
**pDsRed-P8-F**	5'-CACCACCTGTTCCTGAGATCTAAGTCGAGCATACCTAAACCT-3'
**pDsRed-P9-F**	5'-CACCACCTGTTCCTGAGATCTTCGAGCATACCTAAACCTCCT-3'
**pDsRed-P10-F**	5'-CACCACCTGTTCCTGAGATCTAGCATACCTAAACCTCCTCCT-3'
**pDsRed-P11-F**	5'-CACCACCTGTTCCTGAGATCTATACCTAAACCTCCTCCTAGC-3'
**pDsRed-P12-F**	5'-CACCACCTGTTCCTGAGATCTCCTAAACCTCCTCCTAGCTAC-3'
**pDsRed-P13-F**	5'-CACCACCTGTTCCTGAGATCTAAACCTCCTCCTAGCTACTAT-3'
**pDsRed-P14-F**	5'-CACCACCTGTTCCTGAGATCTCCTCCTCCTAGCTACTATGTA-3'
