# Supplementary material for: Identification of a New Conserved Antigenic Epitope by Specific Monoclonal Antibodies Targeting the African Swine Fever Virus Capsid Protein p17
Source: Vet Sci. 2024 Dec 13;11(12):650. doi: 10.3390/vetsci11120650 (PMC11680328; doi:10.3390/vetsci11120650)
Supplement: Supplementary file 1 [file vetsci-11-00650-s001.zip › Supplementary Figures.pdf]

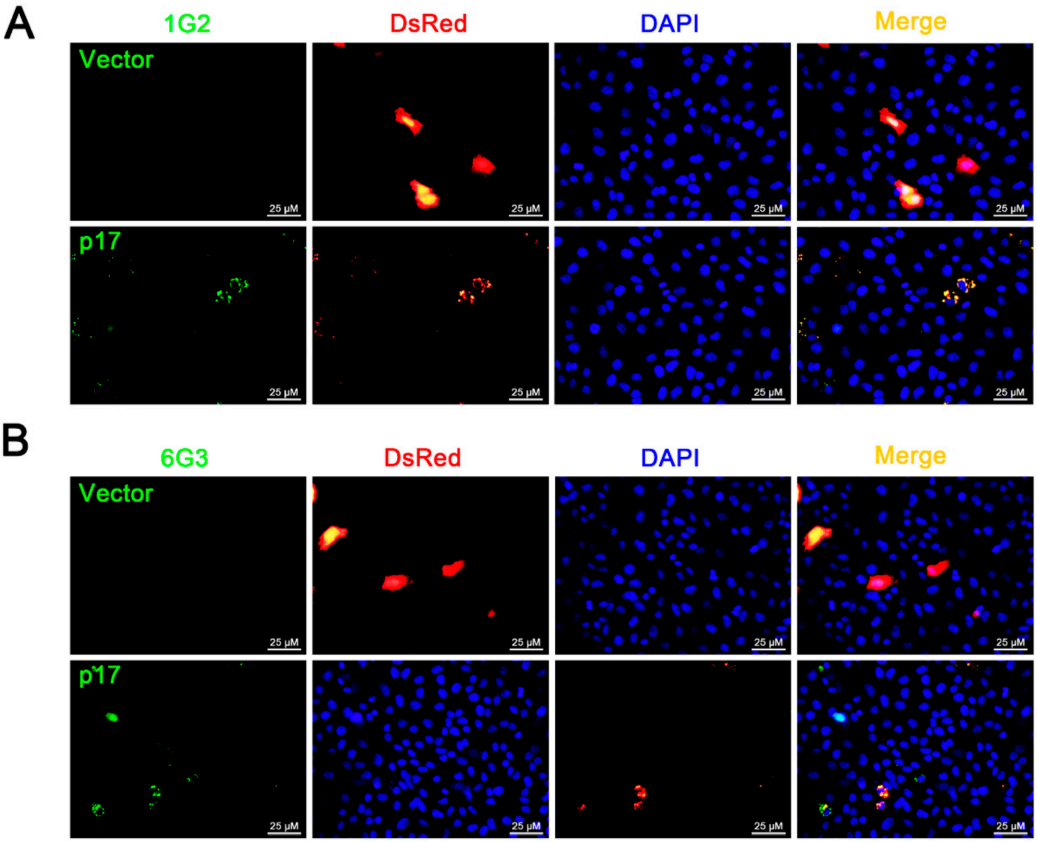

Supplementary Figure 1

Figure S1. Analysis of specific reactivity of p17 mAbs by Immunofluorescence. 3D4/21 cells were transfected pDsRed-p17 (1μg/mL) and pDsRed-C1 vector, respectively. Cells were fixed at 24 h post-transfection and stained with 1G2 mAbs (**A**) and 6G3 (**B**), together with Goat anti-mouse IgG H&L Alexa Fluor 488. Cellular nuclei were counterstained with DAPI. The reactivity of p17 with mAbs was visualized by fluorescence microscopy.

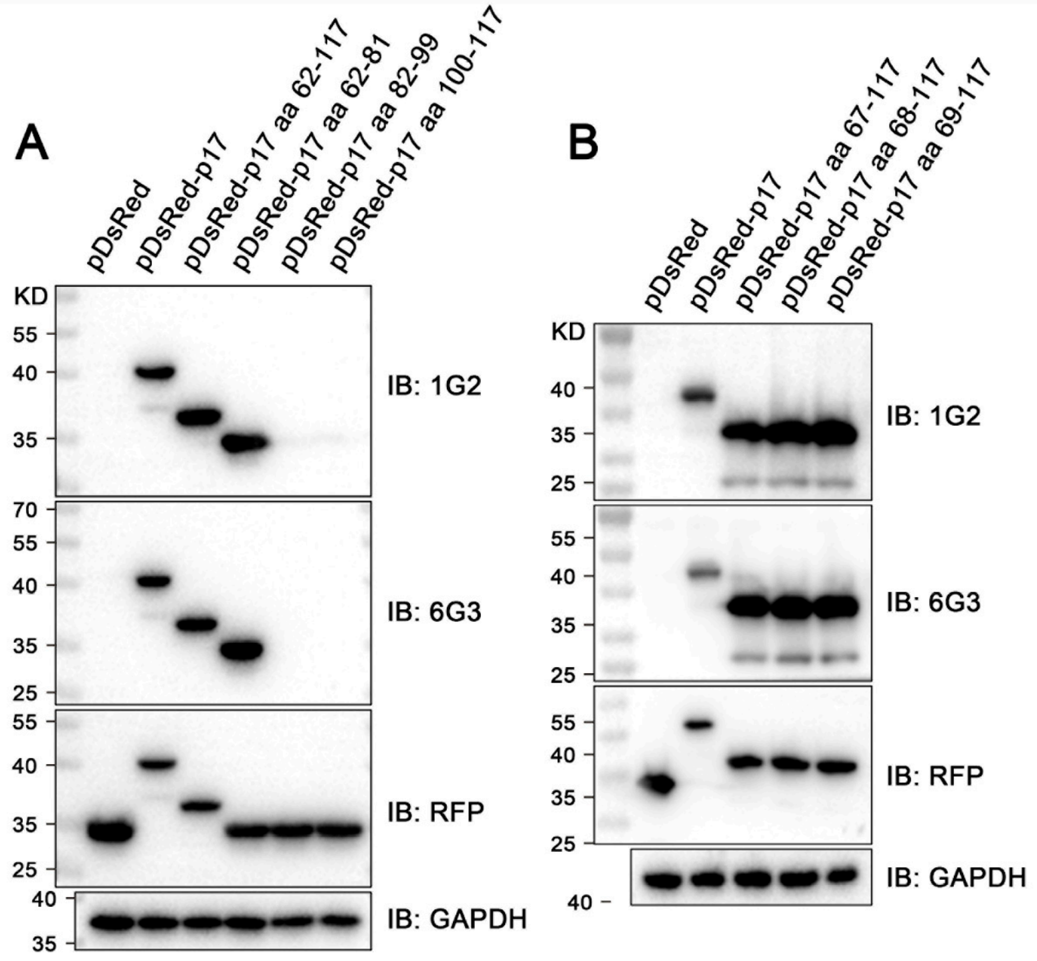

Supplementary Fig 2

Figure S2. The reactivity of different p17 truncated mutants with p17 monoclonal antibodies.

Western blotting analysis of the truncated p17 mutants as indicated in (**A**) and (**B**) with both p17 mAbs 1G2 and 6G3. All mutants were also examined for tag RFP expression using anti-DsRed antibody. The aa is an abbreviation for amino acid.

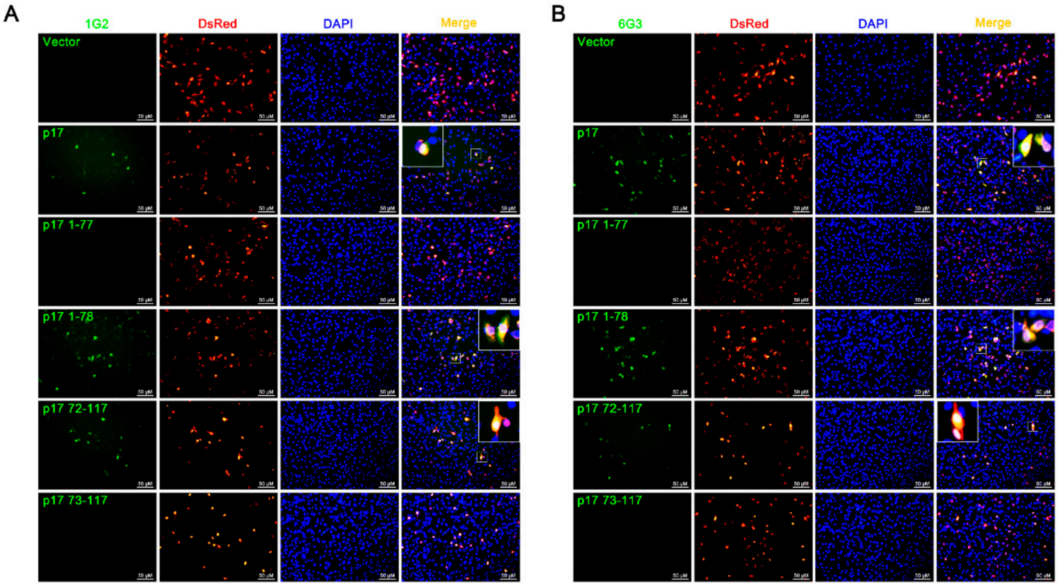

Supplementary Fig 3

Figure S3. Immunofluorescence identification of antigenic epitope recognized by p17 mAbs.

3D4/21 cells were transfected pDsRed-p17 (1μg/mL) and mutants, respectively. Cells were fixed at 24 h post-transfection and stained with mAbs 1G2 (**A**) or 6G3 (**B**), together with Goat anti-mouse IgG H&L Alexa Fluor 488. Cellular nuclei were counterstained with DAPI. The reactivity of p17 mutants with mAbs were observed by fluorescence microscopy. The boxed areas of merged images were magnified and placed on the upright or upleft corners. The p17 and p17 1-78 are mainly expressed in cytoplasm, whereas the p17 72-117 is expressed in whole cells.
