# Supplementary figures and images for: Identification of a New Conserved Antigenic Epitope by Specific Monoclonal Antibodies Targeting the African Swine Fever Virus Capsid Protein p17
Source: Vet Sci. 2024 Dec 13;11(12):650. doi: 10.3390/vetsci11120650 (PMC11680328; doi:10.3390/vetsci11120650)

## Figure 3

A

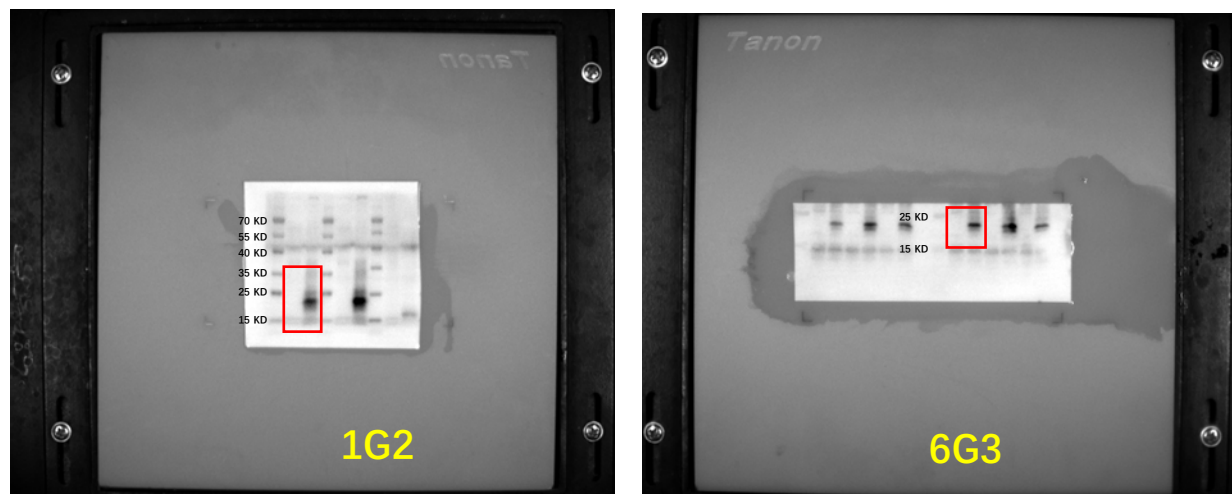

B

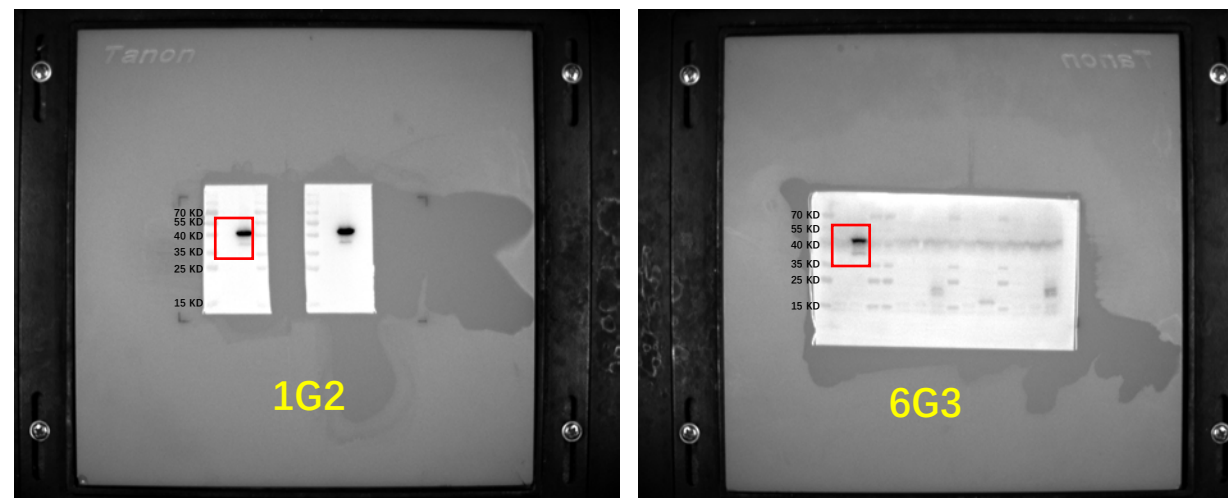

C

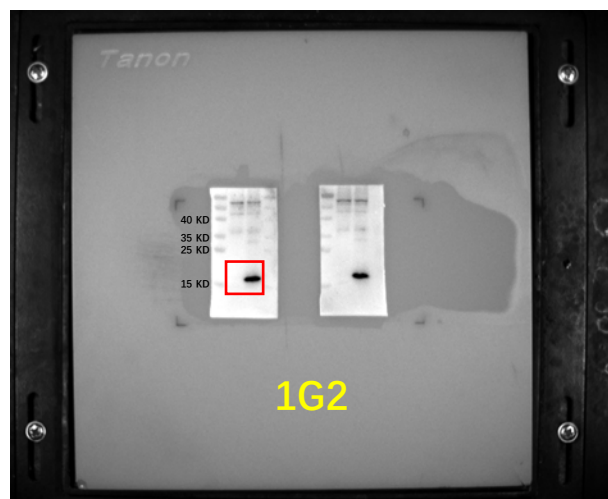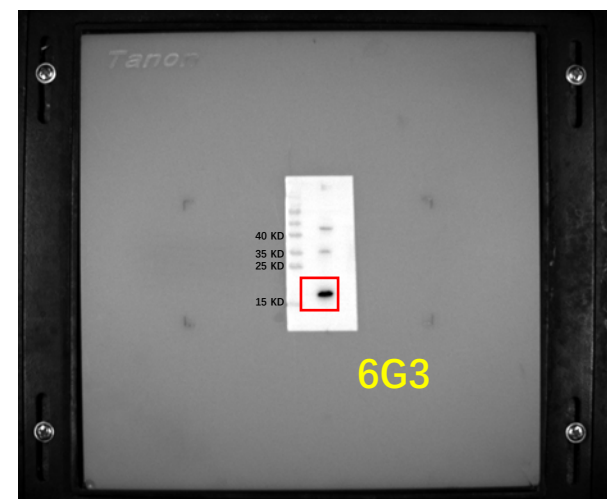

D

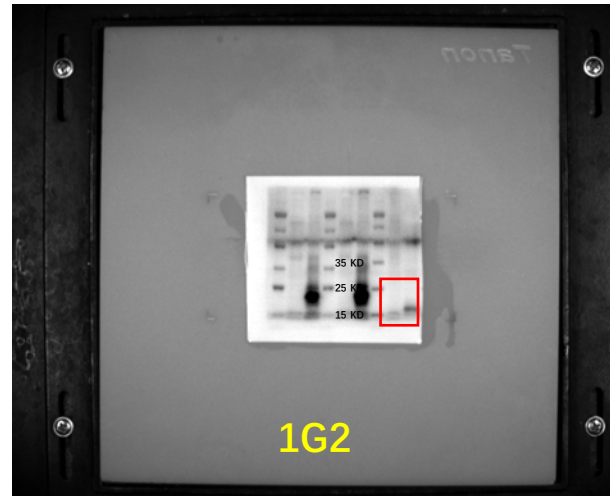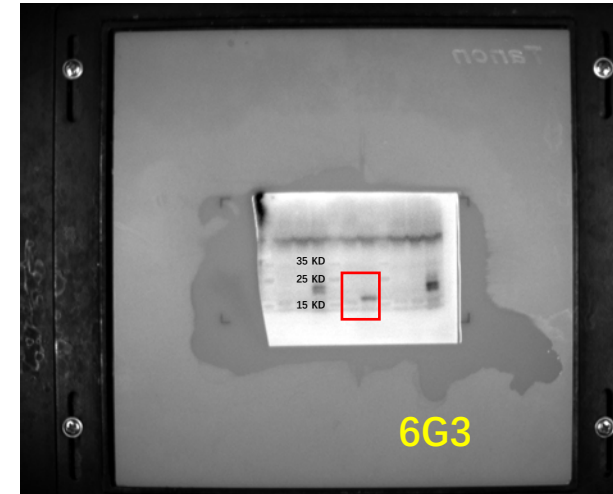

E

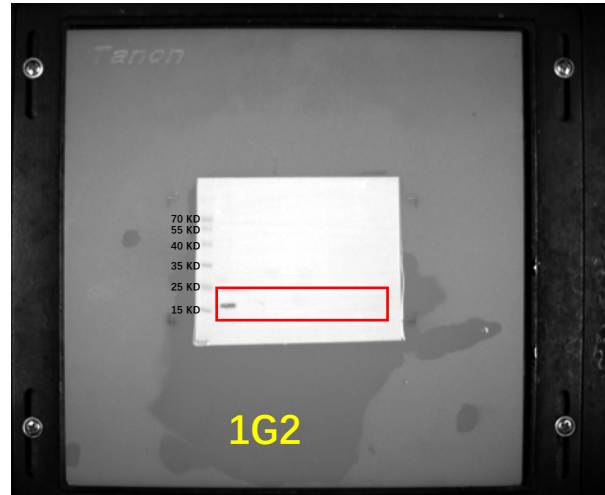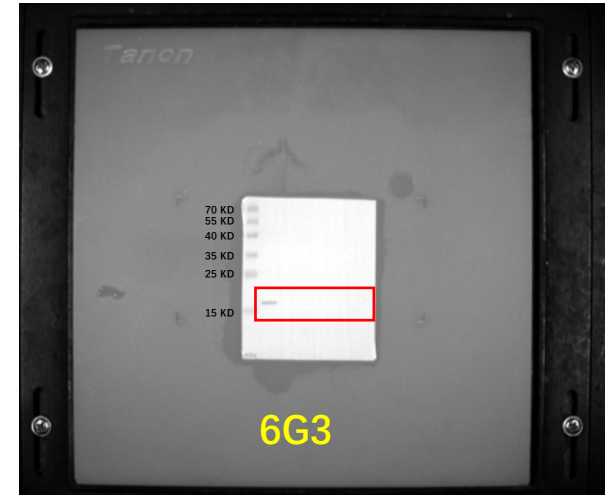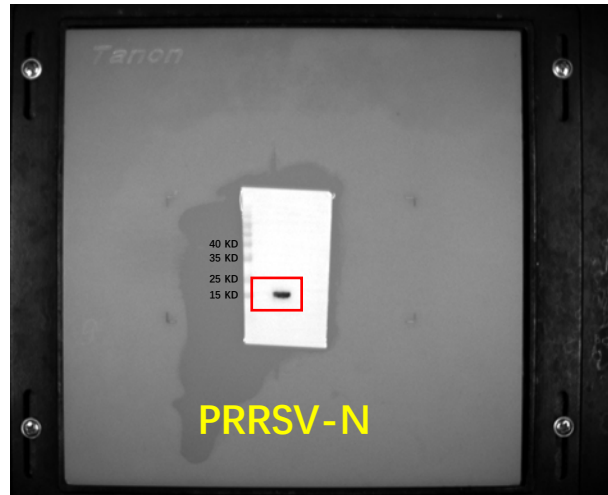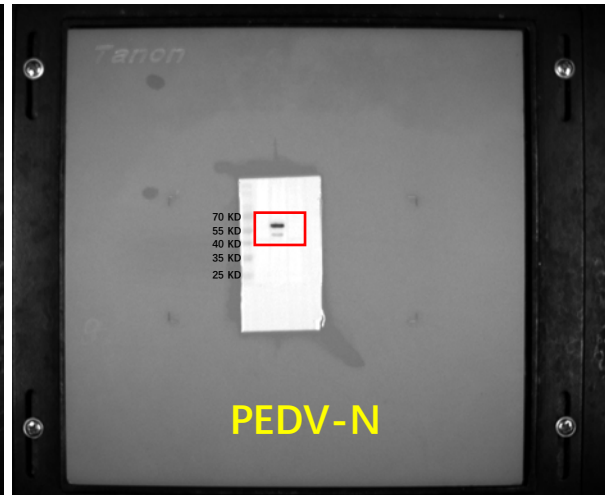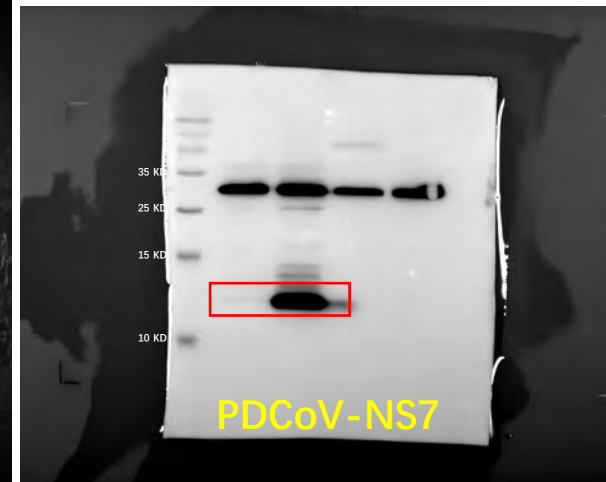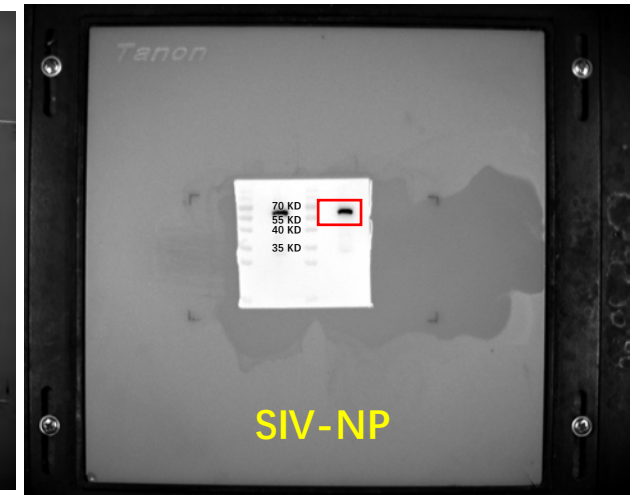

## Figure 5

B

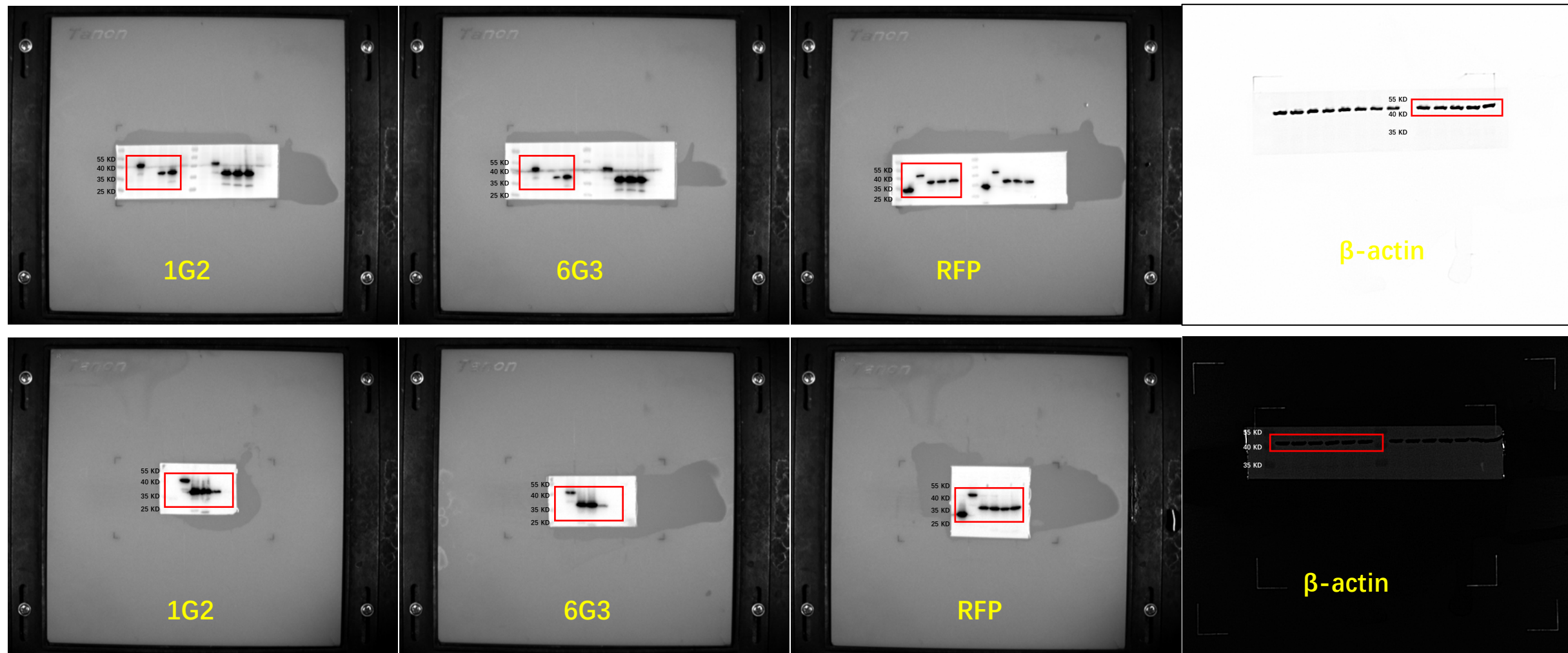

**Figure S3**

A

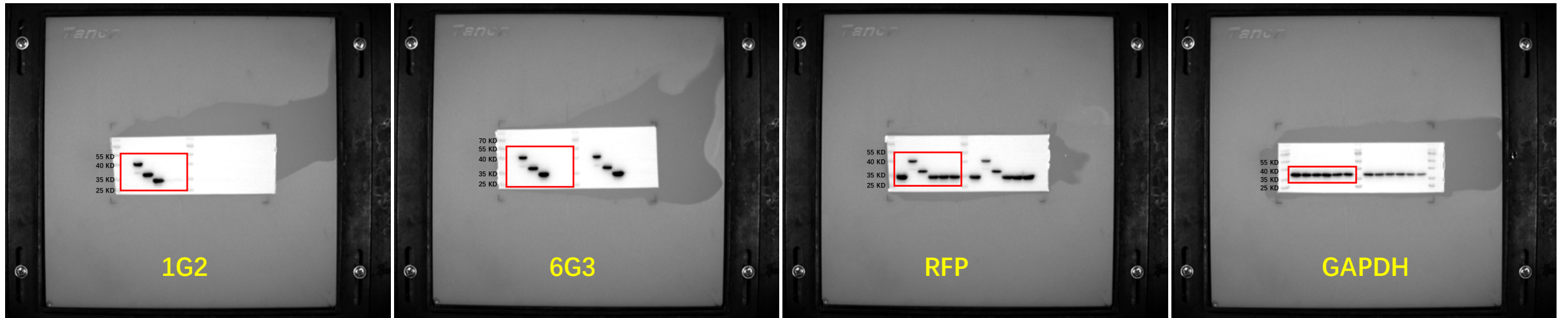

B

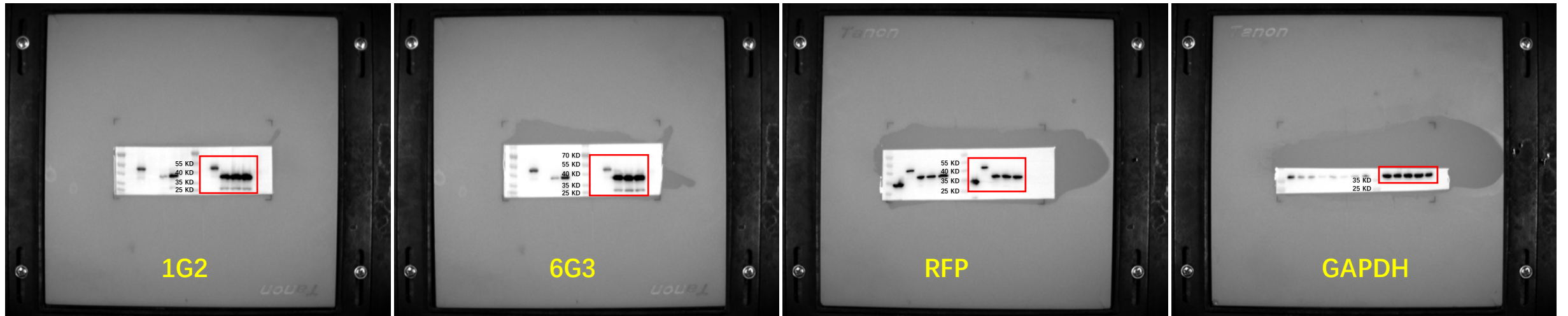

Supplement: Supplementary file 1 [file vetsci-11-00650-s001.zip › WB original data.pdf]
